# Bedaquiline safety, efficacy, utilization and emergence of resistance following treatment of multidrug-resistant tuberculosis patients in South Africa: a retrospective cohort analysis

**DOI:** 10.1186/s12879-022-07861-x

**Published:** 2022-11-21

**Authors:** Helen Pai, Norbert Ndjeka, Lawrence Mbuagbaw, Koné Kaniga, Eileen Birmingham, Gary Mao, Lori Alquier, Kourtney Davis, Arianne Bodard, Abeda Williams, Magalie Van Tongel, Florence Thoret-Bauchet, Shaheed V. Omar, Nyasha Bakare

**Affiliations:** 1 Janssen Research & Development, LLC, Raritan, NJ USA; 2 National TB Programme, South African National Department of Health, Pretoria, South Africa; 3 Department of Health Research Methods, Evidence and Impact, McMaster University, Hamilton, ON Canada; 4 Janssen Research & Development, LLC, Titusville, NJ USA; 5 Janssen Pharmaceutica NV, Beerse, Belgium; 6 Janssen Pharmaceutica (Pty.) Ltd, Johannesburg, South Africa; 7 Janssen-Cilag, Issy-les-Moulineaux, France; 8 Centre for Tuberculosis, National Institute for Communicable Diseases/National Health Laboratory Services, Johannesburg, South Africa; 9 Janssen Global Public Health, Janssen Research & Development, LLC, Titusville, NJ USA

**Keywords:** Bedaquiline/TMC207, DR-TB, South Africa, Registry, Safety/tolerability, WHO treatment outcome, Drug utilization, Resistance, Retrospective cohort study, Tuberculosis, Multidrug-resistant

## Abstract

**Background:**

This retrospective cohort study assessed benefits and risks of bedaquiline treatment in multidrug-resistant-tuberculosis (MDR-TB) combination therapy by evaluating safety, effectiveness, drug utilization and emergence of resistance to bedaquiline.

**Methods:**

Data were extracted from a register of South African drug-resistant-tuberculosis (DR-TB) patients (Electronic DR-TB Register [EDRWeb]) for newly diagnosed patients with MDR-TB (including pre-extensively drug-resistant [XDR]-TB and XDR-TB and excluding rifampicin-mono-resistant [RR]-TB, as these patients are by definition not multidrug-resistant), receiving either a bedaquiline-containing or non-bedaquiline-containing regimen, at 14 sites in South Africa. Total duration of treatment and follow-up was up to 30 months, including 6 months’ bedaquiline treatment. WHO treatment outcomes within 6 months after end-of-treatment were assessed in both patient groups. Longer term mortality (up to 30 months from treatment start) was evaluated through matching to the South African National Vital Statistics Register. Multivariable Cox proportional hazards analyses were used to predict association between receiving a bedaquiline-containing regimen and treatment outcome.

**Results:**

Data were extracted from EDRWeb for 5981 MDR-TB patients (N = 3747 bedaquiline-treated; N = 2234 non-bedaquiline-treated) who initiated treatment between 2015 and 2017, of whom 40.7% versus 80.6% had MDR-TB. More bedaquiline-treated than non-bedaquiline-treated patients had pre-XDR-TB (27.7% versus 9.5%) and XDR-TB (31.5% versus 9.9%) per pre-2021 WHO definitions. Most patients with treatment duration data (94.3%) received bedaquiline for 6 months. Treatment success (per pre-2021 WHO definitions) was achieved in 66.9% of bedaquiline-treated and 49.4% of non-bedaquiline-treated patients. Death was reported in fewer bedaquiline-treated (15.4%) than non-bedaquiline-treated (25.6%) patients. Bedaquiline-treated patients had increased likelihood of treatment success and decreased risk of mortality versus non-bedaquiline-treated patients. In patients with evaluable drug susceptibility testing data, 3.5% of bedaquiline-susceptible isolates at baseline acquired phenotypic resistance. Few patients reported bedaquiline-related treatment-emergent adverse events (TEAEs) (1.8%), TEAE-related bedaquiline discontinuations (1.4%) and QTcF values > 500 ms (2.5%) during treatment.

**Conclusion:**

Data from this large cohort of South African patients with MDR-TB showed treatment with bedaquiline-containing regimens was associated with survival and effectiveness benefit compared with non-bedaquiline-containing regimens. No new safety signals were detected. These data are consistent with the positive risk–benefit profile of bedaquiline and warrant continued implementation in combination therapy for MDR-TB treatment.

**Supplementary Information:**

The online version contains supplementary material available at 10.1186/s12879-022-07861-x.

## Introduction

Tuberculosis (TB) is a significant cause of mortality and morbidity worldwide [1]. A total of 5.8 million cases were newly diagnosed and reported in 2020, which was an 18% drop compared with the 7.1 million cases reported in 2019, caused by disruptions due to the COVID-19 pandemic [1]. Consequently, there was an increase in the number of TB-related deaths from 1.2 million in 2019 to 1.3 million in 2020 [1]. Geographically in 2020, most cases of TB occurred in Southeast Asia (43% of cases) and Africa (25%) [1].

Drug-resistant TB (DR-TB) remains a significant challenge for the global control of TB. Globally, the burden of rifampicin-mono-resistant-TB (RR-TB) or multidrug-resistant (MDR)-TB, defined as *Mycobacterium tuberculosis* strains that are resistant to at least isoniazid and rifampicin, has been stable over the last 10 years, with an estimated 3–4% of new TB cases and 18–21% of previously treated cases being diagnosed as RR- or MDR-TB [1].

Despite improvements in testing, detection, and treatment, only 59% of patients who developed MDR/RR-TB were treated successfully in 2018 [1]. Although MDR-TB is curable, patients cannot be adequately treated with current standard short-course therapy [2, 3]. Therapy for MDR-TB has traditionally required up to 2 years of treatment with older, second-line anti-TB drugs that are mainly bacteriostatic with an unfavorable side-effect profile [4].

Given the emergence of significant drug resistance and low treatment success rates, the introduction of new antibiotics and treatment regimens is vital for the control of this disease. Bedaquiline (SIRTURO®) is a diarylquinoline antimycobacterial agent, with a novel mode of action to inhibit ATP synthase, that has bactericidal and sterilizing properties [5]. Several Phase 2 and real-world studies have demonstrated the effectiveness of bedaquiline-based regimens in the treatment of MDR-TB [6–17], with a positive association with treatment outcomes and reduced mortality [17, 18] and potential for shortening treatment duration [19]. However, these previous studies were either based on smaller cohorts including pre-XDR- or XDR-TB patients only [8, 9], were single-center studies [14, 16], or assessed specific cardiac safety outcomes only [15, 16]. Bedaquiline has received regulatory approval, or accelerated or conditional approval, for use in over 70 countries, including South Africa in October 2014, and has been included in the World Health Organization (WHO) 2020 consolidated guidelines for the treatment of MDR-TB [20], both as a Group A medicine to be prioritized in longer treatment regimens and for use in a standardized all-oral shorter regimen.

A post-marketing requirement for the accelerated approval of SIRTURO® by the United States Food and Drug Administration (FDA) included the development of a patient registry for bedaquiline-treated patients. Additionally, the 2013 WHO interim policy guidance on the use of bedaquiline in the treatment of MDR-TB [21] identified the need for collection of additional data from larger patient populations beyond data provided from the bedaquiline Phase 2 clinical development program.

South Africa is among the countries with a high burden of MDR-TB and high MDR-TB mortality rates [22]. In 2019, 3.4% and 7.1% of TB cases, respectively, were RR-TB or MDR-TB [22]. In South Africa, bedaquiline was rolled out from December 2012 through the national Bedaquiline Clinical Access Programme (BCAP) to allow MDR-TB patients with limited treatment options safe access to this drug, with subsequent national rollout from January 2017. An existing national web-based Electronic Drug-Resistant Tuberculosis Register (EDRWeb) system for routine data collection for South African DR-TB patients, owned by the South African National Department of Health (NDoH), served as the data source to further assess the risk–benefit profile of bedaquiline in MDR-TB combination therapy and adherence to WHO guidance on MDR-TB treatment. Longer term mortality was also evaluated through matching to the South African National Vital Statistics Register. In a Phase 2b study in patients with MDR-TB, a higher proportion of deaths was observed in the bedaquiline group than in the control group, with no causal pattern evident and the median time to death was 49 weeks after the end of bedaquiline treatment [6]. Therefore, the registry follow-up period was for up to 30 months after treatment start.

Here we report the safety, effectiveness, drug utilization and emergence of resistance associated with the use of bedaquiline in the treatment of MDR-TB using data from EDRWeb.

## Methods

### The South African registry

All patients starting DR-TB treatment in South Africa were registered in EDRWeb and the final dataset for analysis was extracted from this, with data on long-term mortality (up to 30 months after the start of treatment) supplemented from the National Vital Statistics Register. Patient demographics was a mandatory field in EDRWeb, but adverse event (AE) reports were optional. However, during the course of the study period (in 2017) EDRWeb was upgraded to include additional data fields to support collection of data for safety reporting and other reports required by the NDoH. Therefore, medical record abstraction was required for certain data fields to ensure completeness in MDR-TB patients initiating treatment in 2015, 2016 and 2017. Data abstraction after the 2017 update of EDRWeb, ensured the data to be as complete as possible. Extracted datasets were shared with an independent third party for registry analysis.

Only data for patients with MDR-TB, excluding RR-TB (as by definition these patients are mono-resistant and not multi-drug resistant) but including extensively drug-resistant TB (XDR-TB) and pre-extensively drug-resistant TB (pre-XDR-TB), were extracted from EDRWeb. Definitions used for XDR-TB and pre-XDR-TB were from pre-2021 WHO guidelines. Specifically, XDR-TB was defined as “TB that is resistant to any fluoroquinolone and to at least one of three second-line injectable drugs (capreomycin, kanamycin and amikacin), in addition to multidrug resistance” [23]. Pre-XDR-TB was defined as “MDR-TB plus fluoroquinolone or any second-line injectable resistance” per the informal definition in use pre-2021 [24]. Data were extracted for patients who started treatment from 2015 to 2017 in four Centers of Excellence located in high-burden provinces accounting for 80% of the MDR-TB disease burden in South Africa (Jose Pearson and Nkqubela in Eastern Cape, King Dinuzulu in KwaZulu-Natal, Sizwe in Gauteng) and 10 primary healthcare facilities in the Khayelitsha subdistrict (in Western Cape).

### Use of bedaquiline in South Africa

The national South African DR-TB treatment guidelines applicable at the start of the data collection period for this study were approved in 2011. Bedaquiline was prescribed to patients with limited treatment options through a limited number of Centers of Excellence from December 2012, prior to its registration, under the BCAP. Bedaquiline was approved as part of combination therapy for treatment of MDR-TB in South Africa in 2014, and bedaquiline and linezolid were introduced in standard regimens for pre-XDR-TB and XDR-TB patients in the 2015 South African guidelines [25] (Additional file 1: Table S1). From 2015 onwards, the use of bedaquiline was extended to all Centers of Excellence and from January 2017, bedaquiline treatment was made possible at the district level. For the period covered by the Registry, only MDR-TB patients were qualified to receive bedaquiline in their treatment regimens per the drug label. The treatment guideline in South Africa was updated in 2018 to consider RR-TB and MDR-TB patients the same in terms of bedaquiline treatment access. None of the registry patients received the short-course regimen.

### Study design and patients

Relevant regulatory and ethical approvals were obtained before initiating this study. Informed consent was not required, as each patient’s consent to treatment was collected in EDRWeb system as part of the existing national TB program. In addition, approval from the local Ethics Committee was obtained prior to extracting data from EDRWeb.

All patients newly diagnosed with MDR-TB (including pre-XDR-TB and XDR-TB and excluding RR-TB) and those newly treated with bedaquiline at participating sites, were eligible for inclusion in the MDR-TB registry. No specific exclusion criteria were applied in this study (so that patients could have been previously treated for TB). A randomly selected subset of MDR-TB patients not exposed to bedaquiline (the comparator cohort) was selected from either patients who started treatment in the 6-month period prior to bedaquiline availability in South Africa or, once bedaquiline became available, from those prescribed a non-bedaquiline-containing regimen. Patient records from EDRWeb were selected to be transferred using a random sampling process, to ensure an almost equal number of bedaquiline-receiving and non-bedaquiline-receiving EDRWeb patient records per facility (stratified by site and treatment).

The decision regarding which MDR-TB treatment regimen (including bedaquiline treatment) was prescribed to the patients was made by the treating physicians based on the patients’ characteristics, disease status and local treatment guidelines. The total treatment and follow-up period was up to 30 months. While treatment was allocated as per local standards and applicable guidelines, a typical MDR-TB treatment duration during the study period was at least 18–24 months, with a 6-month follow-up period after treatment completion. Bedaquiline treatment was generally recommended for a 6-month duration (as per approved label). Data were collected up to 24 months after the last bedaquiline dose unless the patient was lost to follow-up or died.

### Study size

Based on sample size estimates, a registry of 1000 bedaquiline-exposed patients per year over 3 years would have 95% power to detect an AE with a relative risk of 2, assuming alpha = 0.05 and a 1:1 ratio of bedaquiline-exposed patients to unexposed patients, for events that occur with a frequency of at least 3%.

### Data collection and analysis

#### Safety data

Patient safety data were collected as per routine clinical practice in South Africa. The frequency and extent of safety reporting could vary by study site, treating physician, and patient. As recommended by the 2013 WHO interim guidelines [21] on the use of bedaquiline, additional safety monitoring (including electrocardiograms [ECGs]) and active pharmacovigilance were established for the implementation of bedaquiline in South Africa.

Data were collected from the registry on all AEs and treatment-emergent AEs (TEAEs), including serious TEAEs (defined as grade 3, 4 and 5 AEs), bedaquiline-related TEAEs, TEAEs that led to the withdrawal of bedaquiline, and all deaths, including the cause of death, when available. Descriptive TEAE summaries are presented by Medical Dictionary for Regulatory Activities (MedDRA) system organ class and preferred term (MedDRA version 23.0). In addition, AEs of special interest (AESIs) for bedaquiline, identified based on the known clinical safety profile of bedaquiline as well as on preclinical data (i.e., not clinically confirmed), were analyzed by probing the database with Standardised MedDRA (version 23.0) Queries (SMQs) for Acute pancreatitis, Rhabdomyolysis/myopathy, Torsades de pointes/QT prolongation, and drug-related hepatic disorders. ECG data were analyzed descriptively.

### WHO treatment outcome and statistical analysis

Treatment outcome (cured, treatment completed, treatment failed, died, lost to follow-up, not evaluated and treatment success [i.e., the sum of cured and treatment completed patients]), as reported in EDRWeb according to standard WHO definitions pre-2021 (Additional file 1: Table S2) [26], within 6 months after end-of-treatment was assessed among bedaquiline- and non-bedaquiline-treated MDR-TB patients. Frequency distribution (proportions and 95% confidence intervals [CIs]) of the treatment outcomes were summarized and analyzed descriptively by DR-TB type and by year of registration in EDRWeb.

As patients were not randomly assigned to the treatment groups, comparisons of outcomes between treatment groups might be confounded by selection bias. Therefore, treatment success was compared between treatment groups using multivariable logistic regression analysis, adjusting for gender, age, province, human immunodeficiency virus (HIV) status, site of TB (pulmonary or extrapulmonary), history of TB treatment, year of patient registration in EDRWeb and DR-TB type, to estimate the odds ratios (ORs) and 95% CIs for the association between the use of a bedaquiline-containing regimen and treatment success.

Missing data was unavoidable due to the retrospective nature of the study. Efforts were made to minimize missing data via medical record abstraction and matching to the National Vital Statistics Register for mortality. Counts of patients with missing data were reported in all analyses where appropriate (Table 1, Additional file 1: Table S3) and were less than 1% for critical variables; therefore, no imputation was performed.

**Table 1:** Baseline demographic and clinical characteristics of registry MDR-TB patients in South Africa

Characteristic, n (%), unless stated otherwise	Bedaquiline N = 3747	No bedaquiline N = 2234	Total N = 5981
Mean age, years (SD)	37.6 (11.9)	34.2 (14.5)	36.3 (13.0)
Age category, years			
< 12	2 (0.1)	159 (7.1)	161 (2.7)
12–17	111 (3.0)	69 (3.1)	180 (3.0)
≥ 18–64	3549 (94.7)	1955 (87.6)	5504 (92.1)
≥ 65	84 (2.2)	49 (2.2)	133 (2.2)
Sex			
Male	2104 (56.2)	1254 (56.1)	3358 (56.1)
Female	1643 (43.8)	980 (43.9)	2623 (43.9)
Resistance category			
MDR-TB	1526 (40.7)	1801 (80.6)	3327 (55.6)
Pre-XDR-TB	1039 (27.7)	212 (9.5)	1251 (20.9)
XDR-TB	1182 (31.5)	221 (9.9)	1403 (23.5)
Patient treatment category			
New	1419 (37.9)	1015 (45.4)	2434 (40.7)
Relapsed	1216 (32.5)	703 (31.5)	1919 (32.1)
Treatment after loss to follow-up	366 (9.8)	258 (11.5)	624 (10.4)
Treatment after failure 1st line drugs^a^	224 (6.0)	124 (5.6)	348 (5.8)
Treatment after failure 2nd line drugs	467 (12.5)	101 (4.5)	568 (9.5)
Other	55 (1.5)	33 (1.5)	88 (1.5)
HIV and ART^b^			
HIV negative	990 (26.4)	713 (31.9)	1703 (28.5)
HIV positive, not on ART	1263 (33.7)	852 (38.1)	2115 (35.4)
HIV positive, on ART	1491 (39.8)	665 (29.8)	2156 (36.0)
Treatment year			
2015	1004 (26.8)	1116 (50.0)	2120 (35.4)
2016	1465 (39.1)	856 (38.3)	2321 (38.8)
2017	1278 (34.1)	262 (11.7)	1540 (25.7)
Indication for MDR-TB treatment^c^			
Pulmonary TB	3702 (98.8)	2180 (97.8)	5882 (98.3)
Extrapulmonary TB	44 (1.2)	49 (2.2)	93 (1.6)
Evidence of cavitary disease on CXR or CT^d^			
Yes	1074 (31)	4 (6.3)	1078 (30.5)
No	2393 (69)	60 (93.8)	2453 (69.5)

*CT* computed tomography, *CXR* chest x-ray, *MDR* multidrug-resistant, *SD* standard deviation, *TB* tuberculosis, *XDR* extensively drug-resistant, p-values are from Chi-squared and t-tests,* HIV* human immunodeficiency and* ART* antiretroviral therapy

^a^For the treatment of drug-susceptible TB

^b^Three bedaquiline-treated patients and four non-bedaquiline-treated patients had unknown/missing data

^c^One bedaquiline-treated patient and five non-bedaquiline-treated patients had missing data

^d^280 BDQ-treated patients and 2170 non-bedaquiline-treated patients had missing data

The primary mortality analysis was based on deaths captured in EDRWeb as the WHO treatment outcome, defined as a patient who died for any reason during treatment, including deaths captured up to 6 months after end-of-treatment. A multivariable Cox proportional hazards model was used to estimate the hazard ratios (HRs) and 95% CIs for the association between the use of a bedaquiline-containing regimen (compared with a non-bedaquiline-containing regimen) and the risk of mortality, adjusting for potential confounders based on the baseline clinical characteristics and patient demographics described above. A subgroup analysis for treatment success and risk of mortality was performed by year of registration in EDRWeb, gender, age, HIV status, previous drug history and DR-TB type.

In addition, propensity score (PS) methods were used to adjust for imbalances in demographic and disease characteristics between the groups using logistic regression to model the probability of receiving bedaquiline treatment conditional on the potential confounding variables measured and available in the data set (gender, age, province, HIV status, site of TB [pulmonary or extrapulmonary], history of TB treatment, year of patient registration in EDRWeb, and DR-TB type). Multiple statistical approaches using the PS were applied in Cox models, including matching, stratification by PS decile, and inverse probability of treatment weighting. These PS statistical approaches were conducted as a sensitivity analysis to the primary analysis method of multivariable Cox proportional hazards regression for the adjusted association between bedaquiline-containing regimens and death.

### Long-term follow-up mortality data

Long-term mortality data (up to 30 months after the start of treatment) were retrieved from the South African National Vital Statistics Register (for patients lost to follow-up, with missing WHO treatment outcome in EDRWeb, or with deaths reported after the WHO treatment outcome). A multivariable Cox proportional hazards model was used to estimate the HRs and 95% CIs for the association between using a bedaquiline-containing regimen (compared with a non-bedaquiline-containing regimen) and the risk of long-term mortality as described above.

### Sputum culture conversion

Primary diagnosis was based on GeneXpert MTB/RIF result of a patient sputum specimen. Only patients with positive sputum cultures were included in the microbiologic outcome. The frequency distribution of sputum culture conversion (defined as two consecutive negative sputum cultures taken at least 30 days apart after treatment initiation) was summarized. Median time to initial sputum culture conversion was estimated using the Kaplan–Meier method.

### Utilization of MDR-TB treatment

Data were collected on dosages, duration, treatment history, medical history and concomitant medications used for conditions other than TB to describe the pattern of utilization of bedaquiline and MDR-TB treatment in South Africa.

### Evaluation of emergence of resistance

Routine susceptibility testing of MDR-TB drugs at baseline was performed according to the local standard of care to assess the frequency distribution of: MDR-TB excluding pre-XDR and XDR (i.e. TB resistant only to isoniazid and rifampicin; MDR-TB_H&R_), pre-XDR-TB due to fluoroquinolones (pre-XDR-TB_FQ_), pre-XDR-TB due to second-line injectables (pre-XDR_INJ_) and XDR-TB (TB resistant to isoniazid, rifampicin, fluoroquinolones and second-line injectables).

Assessment of emergence of resistance to bedaquiline was performed based on data from the comprehensive National Drug Resistance Surveillance Program and conducted by the National Institute for Communicable Diseases (NICD) and included: bedaquiline drug susceptibility testing (DST) at baseline and post-baseline, when available; and patient outcomes from EDRWeb (culture conversion and treatment success) in patients with bedaquiline-resistant isolates at baseline. Bedaquiline resistance was tested by either broth microdilution minimal inhibitory concentration (MIC) and interpreted as MIC ≥ 0.25 μg/ml or mycobacteria growth indicator tube at the critical concentration of 1 μg/ml. Patients in EDRWeb registry were identified in the NICD database by manual matching of identification numbers.

## Results

### Study population

#### Patient disposition

Data was extracted from EDRWeb for 5981 patients with MDR-TB, exposed to at least one dose of the bedaquiline-containing (N = 3747) or non-bedaquiline-containing regimen (N = 2234) (safety population) at 14 study sites in South Africa between 2015 and 2017. Of the 5981 patients, 5970 (99.8%) patients had baseline and at least one post-baseline WHO treatment outcome and/or microbiological assessment (evaluable population). Eleven patients were excluded from the evaluable population as they were still on treatment (four bedaquiline-treated patients) or had a missing treatment outcome (four bedaquiline-treated patients and three non-bedaquiline-treated patients) at the time of data extraction. Full details of the patient disposition in the South African Registry are presented in Additional file 1: Table S3.

Mean (standard deviation [SD]) estimated duration of registry follow-up (estimated from treatment start to treatment outcome in EDRWeb) was 18.6 (7.5) months for the bedaquiline-treated and 14.8 (9.1) months for non-bedaquiline-treated patients. The total person-time follow-up was 3775 and 1681 person-years, respectively.

Patients’ baseline demographic characteristics (Table 1) were generally similar in the bedaquiline-treated and non-bedaquiline-treated patients, except for a lower proportion of children aged < 12 years in the bedaquiline-treated group (0.1%; lowest age 9 years) than in the non-bedaquiline-treated group (7.1%; lowest age < 1 year), which is to be expected because bedaquiline was not approved for use in children in South Africa during the years of the study.

Most patients (98.3% [5882/5981]) had pulmonary MDR-TB. There was a higher proportion of patients with pre-XDR-TB and XDR-TB in the bedaquiline-treated group than in the non-bedaquiline-treated group (27.7% [1039/3747] and 31.5% [1182/3747] versus 9.5% [212/2234] and 9.9% [221/2234], respectively). Most patients were either newly diagnosed with MDR-TB (37.9% [1419/3747] and 45.4% [1015/2234] for bedaquiline-treated and non-bedaquiline-treated patients, respectively) or had relapsed (32.5% [1216/3747] and 31.5% [703/2234], respectively).

In total, 2754 (73.5%) bedaquiline-treated patients were HIV-positive (of whom 1491 [39.8%] were on antiretroviral therapy [ART]) and 1517 (67.9%) non-bedaquiline-treated patients were HIV-positive (of whom 665 [29.8%] were on ART).

### Treatment information and bedaquiline drug utilization

The most common reasons for starting bedaquiline treatment were because of toxicity of a previous TB regimen (42.5% [1484/3747]), pre-XDR-TB infection (31.3% [1093/3747]), XDR-TB infection (25.1% [875/3747]) and presence of a resistance mutation (15.2% [532/3747]). Reasons were not mutually exclusive. Mean time to bedaquiline treatment initiation was 5 weeks (SD: 11.5). The total duration of prescribed bedaquiline treatment was 6 months in most (94.3% [2900/3076]) bedaquiline-treated patients.

The most frequently used (> 50% of patients) other MDR-TB drugs in the treatment regimen for bedaquiline-treated patients were pyrazinamide (98.3% [3683/3747]), levofloxacin (91.0% [3411/3747]), terizidone (87.5% [3280/3747]), linezolid (78.7% [2949/3747]), clofazimine (69.7% [2613/3747]), ethionamide (64.3% [2409/3747]) and moxifloxacin (52.6% [1971/3747]), and for non-bedaquiline-treated patients were pyrazinamide (97.5% [2179/2234]), terizidone (93.1% [2079/2234]), moxifloxacin (86.9% [1942/2234]), ethionamide (81.5% [1820/2234]) and kanamycin (64.7% [1445/2234]). A higher proportion of bedaquiline-treated than non-bedaquiline treated patients also received concomitant treatment with linezolid (78.7% [2949/3747] versus 7.1% [158/2234], respectively), clofazimine (69.7% [2613/3747] versus 18.0% [403/2234]) or levofloxacin (91.0% [3411/3747] versus 12.9% [288/2234]). A lower proportion of bedaquiline-treated patients received concomitant treatment with injectable agents, amikacin (3.1% [115/3747] versus 13.4% [300/2234]), kanamycin (34.9% [1307/3747] versus 64.7% [1445/2234]) and moxifloxacin (52.6% [1971/3747] versus 86.9% [1942/2234]).

### Concomitant medications

Use of any concomitant medications during MDR-TB treatment was reported for 4784/5981 (80.0%) patients overall: 3263/3747 (87.1%) bedaquiline-treated and 1521/2234 (68.1%) non-bedaquiline-treated patients. The use of concomitant medications in selected medication classes during MDR-TB treatment was reported for 1139/5981 (19.0%) patients and included antiretrovirals (17.6% [1054/5981]), antidepressants (1.8% [110/5981]), cardiovascular medications (1.8% [108/5981]), and antidiabetics (1.0% [59/5981]). There were no notable differences in selected concomitant medication use between the bedaquiline-treated and non-bedaquiline-treated groups.

### Baseline drug susceptibility

Baseline DST for 3172 evaluable bedaquiline-treated and 1567 evaluable non-bedaquiline-treated patients showed that four (0.1%) and 0, respectively, were infected with an MDR-TB isolates that showed resistance to bedaquiline at baseline.

Baseline DST for other MDR-TB drugs showed 1262/3172 (39.8%) and 1200/1567 (76.6%) patients, respectively, were infected with MDR-TB_H&R_; 417 (13.1%) and 77 (4.9%) were infected with pre-XDR-TB_FQ_; 483 (15.2%) and 108 (6.9%) were infected with pre-XDR-TB_INJ_; and 1010 (31.8%) and 182 (11.6%) were infected with XDR-TB. The highest frequency of resistance in patients overall (> 20%) was to rifampicin (90.4% [4282/4739]), isoniazid (86.1% [4080/4739]), ofloxacin (33.3% [1577/4739]), capreomycin (30.1% [1428/4739]) and amikacin (22.4% [1.060/4739]).

### Bedaquiline effectiveness and development of resistance

#### WHO treatment outcome

Treatment success was achieved in 2501/3739 (66.9%) bedaquiline-treated patients versus 1102/2231(49.4%) non-bedaquiline-treated patients, with 1934/3739 (51.7%) versus 850/2231 (38.1%) patients, respectively, considered cured, and 567/3739 (15.2%) versus 252/2231 (11.3%) patients, respectively, having completed treatment (Table 2). Death was reported in 577/3739 (15.4%) and 572/2231(25.6%) bedaquiline-treated and non-bedaquiline-treated patients, respectively.

**Table 2:** WHO treatment outcomes by bedaquiline treatment in MDR-TB patients in South Africa

WHO outcomes, n (%), and 95% CIs^a^	Bedaquiline N = 3739^b^	No bedaquiline N = 2231^b^
Cured	1934 (51.7) 50.1, 53.3	850 (38.1) 36.1, 40.2
Treatment completed	567 (15.2) 14.0, 16.4	252 (11.3) 10.0, 12.7
Died	577 (15.4) 14.3, 16.6	572 (25.6) 23.8, 27.5
Lost to follow-up	553 (14.8) 13.7, 16.0	465 (20.8) 19.2, 22.6
Treatment failed	108 (2.9) 2.4, 3.5	92 (4.1) 3.3, 5.0
Treatment success (cured + treatment completed)	2501 (66.9) 65.4, 68.4	1102 (49.4) 47.5, 51.4

^a^The 95% CI is based on the Clopper-Pearson exact binomial method

^b^The 11 patients excluded from the evaluable population were 4 patients that were still on treatment (all bedaquiline-treated) and 7 patients with missing treatment outcome (4 bedaquiline-treated patients and 3 non-bedaquiline-treated patients)

**Fig. 1:**
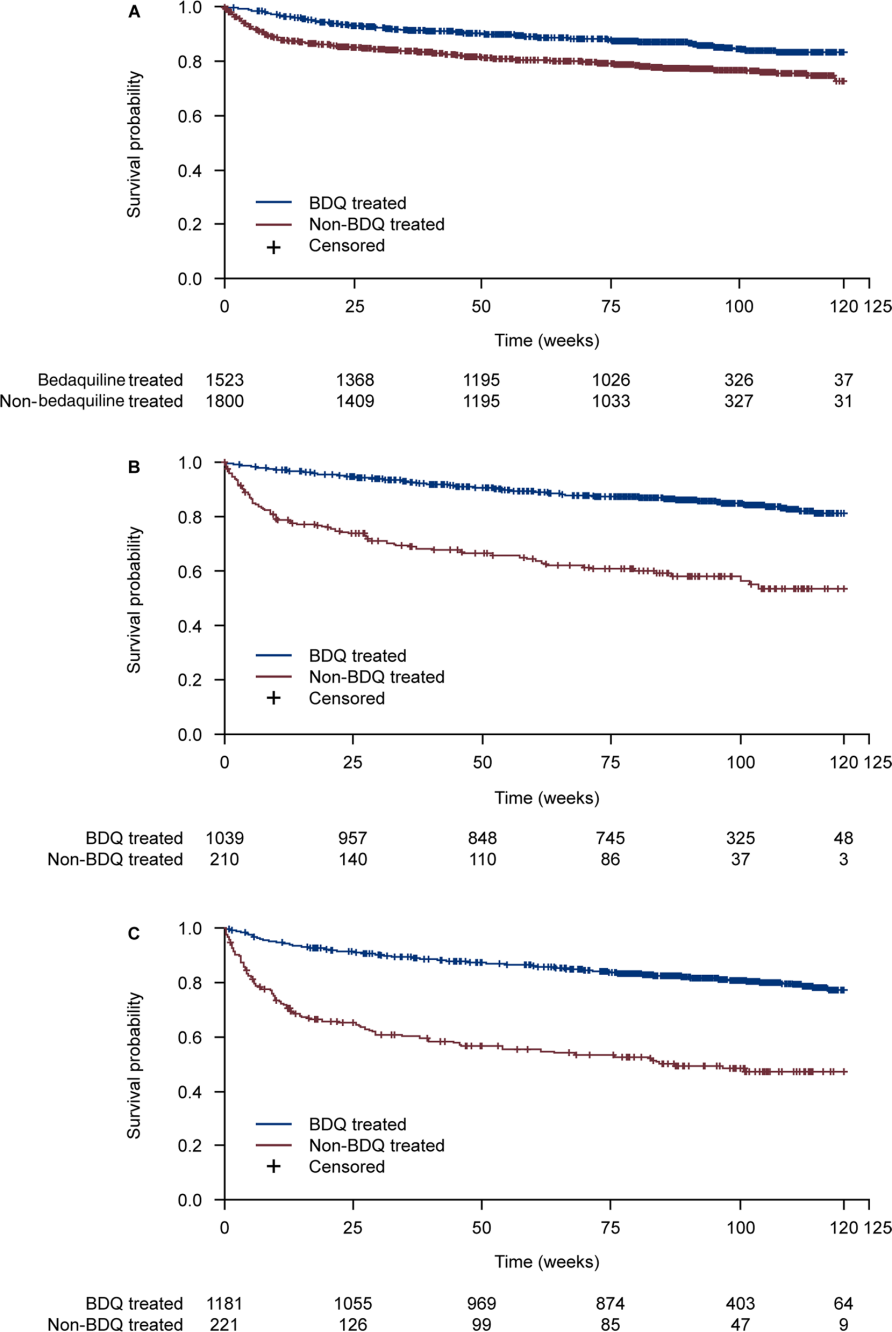
Kaplan–Meier survival curve for deaths defined per WHO treatment outcome for **A** MDR-TB-infected patients, **B** pre-XDR-TB-infected patients and **C** XDR-TB-infected patients in South Africa treated with bedaquiline- or non-bedaquiline-containing regimens

The results for the proportion of patients who had treatment success or who died by DR-TB subtypes and by year of patient registration in EDRWeb (data not shown) were consistent with the overall WHO treatment outcome and mortality results. Numerically higher proportions of bedaquiline- versus non-bedaquiline-treated patients with MDR-TB, pre-XDR-TB and XDR-TB achieved treatment success (Table 3). Figure 1 shows the Kaplan–Meier survival curves for death, defined per WHO treatment outcome, by treatment group for patients with MDR-TB, pre-XDR-TB, and XDR-TB.

**Table 3:** WHO treatment outcomes by bedaquiline treatment and DR-TB type in MDR-TB patients in South Africa

WHO outcomes, n (%)	MDR N = 3323		Pre-XDR N = 1246		XDR N = 1401	
	Bedaquiline N = 1523	No bedaquiline N = 1800	Bedaquiline N = 1036	No bedaquiline N = 210	Bedaquiline N = 1180	No bedaquiline N = 221
Cured	778 (51.1)	753 (41.8)	515 (49.7)	49 (23.3)	641 (54.3)	48 (21.7)
Treatment completed	251 (16.5)	215 (11.9)	158 (15.3)	17 (8.1)	158 (13.4)	20 (9.0)
Died	205 (13.5)	385 (21.4)	148 (14.3)	82 (39.0)	224 (19.0)	105 (47.5)
Lost to follow-up	254 (16.7)	397 (22.1)	182 (17.6)	49 (23.3)	117 (9.9)	19 (8.6)
Treatment failed	35 (2.3)	50 (2.8)	33 (3.2)	13 (6.2)	40 (3.4)	29 (13.1)
Treatment success (cured + treatment completed)	1029 (67.6)	968 (53.8)	673 (65.0)	66 (31.4)	799 (67.7)	68 (30.8)

Multivariable logistic regression analysis, adjusting for gender, age, province, HIV status, type of TB (pulmonary and extrapulmonary MDR-TB), previous drug history, year of patient registration in EDRWeb, and DR-TB type (MDR-TB, pre-XDR-TB, and XDR-TB), showed that treatment with a bedaquiline-containing regimen was associated with increased likelihood of treatment success compared with an MDR-TB regimen not containing bedaquiline (OR 2.57; 95% CI: 2.26, 2.93) (Additional file 1: Fig. S1a). In general, a consistent beneficial effect of a bedaquiline-containing regimen on treatment success was observed across subgroups (Additional file 1: Fig. S1a).

Multivariable Cox proportional hazards analysis, adjusting for the same factors described above, showed that treatment with a bedaquiline-containing regimen was associated with decreased risk of mortality (defined per WHO treatment outcome) compared with an MDR-TB regimen not containing bedaquiline (HR 0.36; 95% CI: 0.31, 0.41) (Additional file 1: Fig. S1b). The effect of a bedaquiline-containing regimen on reducing the risk of mortality was also consistent across the various subgroups (Additional file 1: Fig. S1b). The results of the various PS statistical approaches from sensitivity analyses were consistent with the findings from the adjusted Cox proportional hazards model, showing a consistent beneficial effect of bedaquiline-containing regimens on the risk of mortality (WHO treatment outcome) (Additional file 1: Table S4).

### Long-term follow-up mortality data

Long-term follow-up mortality data (Additional file 1: Fig. S2) showed a similar trend to mortality defined per WHO treatment outcome (EDRWeb data only, end of treatment). Mortality at 30 months (130 weeks) was reported in 721/3743 (19.3%) bedaquiline-treated and 654/2231 (29.3%) non-bedaquiline-treated patients, with the multivariable Cox proportional hazards analysis showing a decreased risk of long-term mortality for bedaquiline-treated compared with non-bedaquiline-treated patients overall (HR 0.43; 95% CI: 0.38, 0.49) and across subgroups (data not shown).

### Sputum culture conversion

At baseline, 2483/2903 (85.5%) bedaquiline-treated and 1358/1692 (80.3%) non-bedaquiline-treated patients had a positive sputum culture result at baseline (Table 4). Of these patients, 2084/2483 (83.9%) and 868/1358 (63.9%), respectively, achieved culture conversion (two consecutive negative cultures at least 30 days apart), with a median time to culture conversion of 102 (95% CI: 98, 106) and 83 (95% CI: 78, 88) days, respectively (Table 4). At end of follow-up, most patients in the evaluable population had a negative smear result (88.9% [3141/3533] of bedaquiline-treated patients and 86.1% [1574/1829] of non-bedaquiline-treated patients) or a negative culture result (83.7% [2685/3207] and 81.2% [1355/1668], respectively).

**Table 4:** Microbiologic results by bedaquiline treatment in MDR-TB patients in South Africa

	Bedaquiline N = 3739^a^	No bedaquiline N = 2231^a^
Sputum culture at baseline		
Positive, n (%)	2483 (85.5)	1358 (80.3)
Negative, n (%)	420 (14.5)	334 (19.7)
Missing, n	836	539
Sputum culture conversion^b^		
To negative, n/N (%)	2084/2483 (83.9)	868/1358 (63.9)
Non-conversion, n/N (%)	399/2483 (16.1)	490/1358 (36.1)
Sputum culture reversion^c^		
To positive, n/N (%)	127/2084 (6.1)	25/868 (2.9)
*Times to sputum culture initial conversion*	*N = 2483*	*N = 1358*
Median, days	102	83
95% CI	98, 106	78, 88

*CI* confidence interval, The 95% CI is based on the Clopper-Pearson exact binomial method

^a^The 11 patients excluded from the evaluable population were 4 patients that were still on treatment (all bedaquiline-treated) and 7 patients with missing treatment outcome (4 bedaquiline-treated patients and 3 non-bedaquiline-treated patients)

^b^Percentage is based on the number who were positive at baseline and their status at two consecutive visits at least 30 days apart: negative (conversion) or positive (non-conversion); Baseline was defined as the most recent result/measurement prior to the first dose

^c^Percentage is based on the number who had a conversion to negative

### Development of resistance to bedaquiline

Based on the NICD analysis, of 383/3747 (10.2%) bedaquiline-treated patients with a valid baseline bedaquiline DST in the database, 19/383 (5.0%) had *M. tuberculosis* isolates that showed resistance to bedaquiline at baseline, including one patient with RR-TB (but who was characterized as having MDR-TB in the registry based on local laboratory DST), four patients with MDR-TB, five with pre-XDR-TB and nine with XDR-TB. None of the 19 bedaquiline-resistant isolates at baseline had an *atpE* resistance-associated variant (RAV). Five of 19 patients had isolates with no efflux pump regulatory gene, *Rv0678* RAVs, nine isolates had insertions leading to a frameshift, and five isolates had a non-synonymous substitution in the *Rv0678* locus. WHO treatment outcome was cured in seven patients, lost to follow-up in five patients, completed treatment in four patients, death in two patients, and one had an unknown outcome.

For the assessment of emerging resistance, of 364 bedaquiline-treated patients infected with *M. tuberculosis* isolates susceptible to bedaquiline at baseline, 142 (39.0%) had at least one post-baseline evaluable sample (≥ 30 days post-baseline) and were included in the analysis. Overall, 5/142 (3.5%) patients had isolates that developed resistance to bedaquiline post-baseline. One patient had MDR-TB, two had pre-XDR-TB and two had XDR-TB at baseline. None of the five isolates with treatment-emergent resistance to bedaquiline had an *atpE* RAV. Four of the five isolates had nucleotide insertions in *Rv0678*, leading to a frameshift and one had a nucleotide deletion. WHO treatment outcomes were ‘lost to follow-up’ in two patients and ‘cured’, ‘death’ and ‘treatment completed’ each in one patient. The majority (66.4% [91/137]) of patients with isolates that did not develop resistance to bedaquiline on treatment had a successful outcome (cured or treatment completed).

### Safety

#### Most frequent TEAEs

The most frequent TEAEs (≥ 10% of patients), by preferred term, were vomiting, arthralgia, rash, hypothyroidism, peripheral neuropathy, ototoxicity, and pain in extremity for bedaquiline-treated patients and ototoxicity and vomiting for non-bedaquiline-treated patients (Table 5).

**Table 5:** TEAEs in bedaquiline- and non-bedaquiline-treated MDR-TB patients in South Africa

Parameter, n (%)	Bedaquiline N = 3747	No bedaquiline N = 2234	Total N = 5981
≥ 1 TEAE	3139 (83.8)	1282 (57.4)	4421 (73.9)
≥ 1 bedaquiline-related TEAE	67 (1.8)	NA	67 (1.1)
≥ 1 TEAE leading to bedaquiline withdrawal	54 (1.4)	NA	NA
≥ 1 serious TEAE	1491 (39.8)	581 (26.0)	2072 (34.6)
TEAEs by preferred term reported in > 10% of patients in either arm			
Vomiting	884 (23.6)	304 (13.6)	1188 (19.9)
Arthralgia	843 (22.5)	198 (8.9)	1041 (17.4)
Rash	783 (20.9)	199 (8.9)	982 (16.4)
Hypothyroidism	669 (17.9)	155 (6.9)	824 (13.8)
Peripheral neuropathy	619 (16.5)	159 (7.1)	778 (13.0)
Ototoxicity	542 (14.5)	421 (18.8)	963 (16.1)
Pain in extremity	500 (13.3)	131 (5.9)	631 (10.6)
Serious TEAEs by preferred term reported in > 2% of patients in either arm			
Ototoxicity	472 (12.6)	317 (14.2)	789 (13.2)
Anemia	190 (5.1)	4 (0.2)	194 (3.2)
Optic neuritis	175 (4.7)	35 (1.6)	210 (3.5)
ECG QT prolonged	109 (2.9)	3 (0.1)	112 (1.9)
Peripheral neuropathy	90 (2.4)	15 (0.7)	105 (1.8)
Decreased hemoglobin	89 (2.4)	2 (0.1)	91 (1.5)
Vomiting	88 (2.3)	27 (1.2)	115 (1.9)
Psychotic disorder	74 (2.0)	32 (1.4)	106 (1.8)

#### Bedaquiline-related TEAEs

Bedaquiline-related TEAEs were reported for 67/3747 (1.8%) bedaquiline-treated patients (Table 5), most frequently ECG QT interval prolongation, reported for 56/3747 (1.5%) patients. Other bedaquiline-related TEAEs (abdominal pain, increased amylase, atrioventricular block, blurred vision, increased blood lactate dehydrogenase, cardiac disorder, cardiotoxicity, abnormal ECG, toxic and peripheral neuropathy) were reported for ≤ 0.1% of patients.

#### TEAEs leading to withdrawal

One or more TEAEs leading to permanent withdrawal of bedaquiline were reported in 54/3747 (1.4%) bedaquiline-treated patients (Table 5), most commonly ECG QT interval prolongation, reported in 45/3747 (1.2%) patients. Other AEs leading to permanent discontinuation of bedaquiline (abdominal pain, increased amylase, atrioventricular block, increased blood lactate dehydrogenase, cardiac disorder, cardiotoxicity, abnormal ECG, toxic nephropathy and peripheral neuropathy) all occurred in only one patient (< 0.1%).

TEAEs leading to permanent discontinuation of any drug in the MDR-TB regimen were recorded in 1399/3747 (37.3%) bedaquiline-treated and 490/2234 (21.9%) non-bedaquiline-treated patients.

#### AESIs

Torsades de pointes/QT prolongation SMQ events were reported for 179/3747 (4.8%) and 5/2234 (0.2%) patients, respectively. All events captured by the Torsades de pointes/QT prolongation SMQ were QT prolongation. There were no reports of Torsades de pointes.

Based on available ECG data, post-baseline abnormal QTcF values > 480–500 ms were reported in 96/2278 (4.2%) bedaquiline-treated and 2/38 (5.3%) non-bedaquiline-treated patients with post-baseline ECG data. Post-baseline QTcF values > 500 ms were observed in 58/2,278 (2.5%) bedaquiline-treated patients and no non-bedaquiline-treated patients. Of the 58 bedaquiline-treated patients, the majority took additional QT-prolonging drugs during their MDR-TB treatment (96.6% [56/58] used levofloxacin, 91.4% [53/58] clofazimine and 46.6% [27/58] moxifloxacin), and 13/58 had a WHO treatment outcome of death.

Drug-related hepatic disorders SMQ events were reported in 75/3747 (2.0%) bedaquiline-treated patients and 35/2234 (1.6%) non-bedaquiline-treated patients, mostly concerning a hepatitis TEAE (32 and 27 cases, respectively) or hepatotoxicity TEAE (22 and two cases, respectively).

Acute pancreatitis SMQ events were reported for 4/3747 (0.1%) bedaquiline-treated and no non-bedaquiline-treated patients; and Rhabdomyolysis/myopathy SMQ for 0 and 1/2234 (< 0.1%) patients, respectively.

#### Serious TEAEs

Serious TEAEs (grade 3–5 severity) were reported in 1491/3747 (39.8%) bedaquiline-treated and 581/2234 (26.0%) non-bedaquiline-treated patients (Table 5). The most common serious TEAEs for bedaquiline-treated patients (reported in ≥ 2.0% of patients) were ototoxicity, anemia, optic neuritis, ECG QT prolonged, peripheral neuropathy, decreased hemoglobin, vomiting and psychotic disorder (Table 5).

## Discussion

This retrospective, observational cohort study using data from the South African national patient register of DR-TB patients showed that treatment success (sum of cured and treatment completed patients) was achieved in 66.9% of bedaquiline-treated and 49.4% of non-bedaquiline-treated patients, and death was reported in 15.4% and 25.6% of patients, respectively. Adjusted multivariable Cox proportional hazards analyses showed that treatment with a bedaquiline-containing regimen was associated with increased likelihood of treatment success and decreased risk of short- and long-term mortality compared with an MDR-TB regimen not containing bedaquiline. Furthermore, a consistent beneficial effect of a bedaquiline-containing regimen on treatment success and mortality risk was observed across the subgroups. However, some subgroups included a limited number of patients, with an imbalance in the proportion of bedaquiline-treated and non-bedaquiline-treated patients, so the results should be interpreted with caution. The majority of TEAEs generally reflected the known safety profile of bedaquiline and background regimen medications.

Bedaquiline was used as prescribed by the physician in this real world, observational study design. The duration of bedaquiline treatment was generally ≤ 6 months (i.e., for 94% of patients), which is in line with the recommended duration in the approved label [27]. Background regimen drugs used in combination with bedaquiline reflected recommendations in local guidelines, particularly regarding the treatment of patients with pre-XDR-TB and XDR-TB.

Survival and effectiveness benefit linked to the use of bedaquiline- compared with non-bedaquiline-based treatment was observed despite more bedaquiline-treated patients having pre-XDR-TB and XDR-TB and HIV co-infection. Factors that could have contributed positively to the observed effectiveness of the bedaquiline- compared with non-bedaquiline-based treatment regimen included (1) bedaquiline-treated patients may have received more careful follow-up and management in line with the 2013 WHO guideline [21] and local South African guidelines [25], and (2) were more likely to receive other new/repurposed drugs such as linezolid and clofazimine. However, the survival and effectiveness benefit for bedaquiline-containing regimens was consistent with previously published data from South Africa. In a prospective follow-up of 272 South African patients with XDR-TB, the 24-month favorable outcome rate was markedly better in patients who received bedaquiline than in those who did not (66.2% vs 13.2%; p < 0.001); 24-month treatment failure was also less frequent with bedaquiline (5.9% vs 26.0%; p < 0.001) [11]. Rate of treatment success in all bedaquiline-treated patients in the present study (66.9%) was also consistent with results from two previous smaller cohort studies including bedaquiline-treated pre-XDR- and XDR-TB patients only (rate of treatment success: 73.0% [9] and 63.6% [14]). An additional retrospective cohort study utilizing data from 1016 South African patients with RR/MDR-TB or XDR-TB who were treated with a bedaquiline-containing regimen in EDRWeb between July 1, 2014, and March 31, 2016 also reported on mortality by linking to the national Vital Statistics Register [17]. Bedaquiline-based treatment regimens were associated with a large reduction in all-cause mortality for patients with RR/MDR-TB (HR 0.35: 95% CI: 0.28, 0.46) and XDR-TB (HR 0.26: 95% CI: 0.18, 0.38) compared with standard regimens [17]. In this interim analysis, not all patients had completed treatment, so the present study supports these observations and provides further data, as 99.8% of patients treated with bedaquiline in the present study have a final outcome. Further, the present study contains data from patients treated more recently (2015 to 2017, compared with 2014 to 2016 in the previous study); the number of patients treated with bedaquiline-containing regimens is more than three times higher than in the previous study, and patients with pre-XDR-TB were included in the present study but excluded in the previous study [17]. It should also be noted that the time to sputum culture conversion in patients receiving bedaquiline was longer than those not receiving bedaquiline (median, 102 versus 83 days). Although the explanation behind this finding is unclear, it may result from the higher proportion of patients receiving bedaquiline who had pre-XDR-TB (27.7% versus 9.5% for those not receiving bedaquiline; pre-2021 WHO definitions) or XDR-TB (31.5% versus 9.9%).

Development of phenotypic resistance to bedaquiline on treatment was low; five of the 142 patients (3.5%) with isolates susceptible to bedaquiline at baseline had isolates that subsequently developed resistance. Most patients (60%; 222/364) were culture negative, presumably due to the bactericidal effect of bedaquiline in the treatment regimens. When the total number of baseline bedaquiline sensitive isolates (n = 222) was taken into account, the phenotypic bedaquiline resistance rate on treatment was 1.4% (5/364). Among the five patients with development of bedaquiline phenotypic resistance, the reported outcomes within 6 months after the end of treatment were: ‘lost to follow-up’ in two patients and ‘cured’, ‘death’, and ‘treatment completed’, each in one patient. All increases in MIC for bedaquiline were explained by mutations in the efflux pump regulatory gene *Rv0678* gene (and not in *atpE* bedaquiline target), as reported previously for bedaquiline [28, 29]. In an observational study, Ismail and colleagues, from the NICD and other collaborating institutes, carried out a cross-sectional and longitudinal analysis of the epidemiology, genetic basis, and treatment outcomes associated with bedaquiline resistance, using data (between 2015 and 2019) from the National Drug Resistance Surveillance Program [30]. Three databases were linked and used in their analysis: the NICD TB laboratory database and the NICD national TB surveillance database that was matched to the EDRWeb. In their analysis, treatment-emergent resistance to bedaquiline occurred in 2.3% of patients, similar to the data presented here, with the majority of cases occurring in patients with pre-XDR-TB or XDR-TB. Phylogenetic analysis showed that in most cases, acquisition of phenotypic resistance was associated with the presence of an *Rv0678* mutation that was not present at baseline, also in agreement with our results.

Some of the most frequently reported adverse drug reactions, vomiting, arthralgia, rash, hypothyroidism, peripheral neuropathy, and pain in extremity, were reported more frequently in bedaquiline-treated than non-bedaquiline-treated patients. The higher incidence in the bedaquiline group for vomiting and arthralgia is not unexpected as they are known AEs for bedaquiline. A higher proportion of bedaquiline-treated than non-bedaquiline-treated patients also received concomitant treatment with linezolid or clofazimine, which may also explain the higher incidences of vomiting and rash, which are known side effects for both drugs, and neurologic and ophthalmologic AEs, known to be associated with linezolid use [31]. Dermatologic conditions are common in patients with HIV and are also a common side effect of ART. The higher incidence of rash in bedaquiline-treated patients may also be due to a higher proportion than non-bedaquiline-treated patients being HIV-positive. Most bedaquiline-treated patients experiencing rash (76.2% [597/783]) were HIV-positive and 334/783 (42.7%) patients were on ART at baseline. There were no reports of bedaquiline-related rash. Most of those patients reporting hypothyroidism also took ethionamide as part of their treatment regimen, for which this event is a known AE [32–34]. Only 1.8% of those patients on bedaquiline-containing regimens had bedaquiline-related TEAEs, most commonly ECG QT interval prolongation, reflecting the known safety profile of bedaquiline. DR-TB drugs regimens used at the time of the study have documented high frequency of AEs associated with them. Therefore, it is unsurprising that serious TEAEs were reported in a relatively high proportion of the patients in each treatment group. However, 1.4% of bedaquiline-treated patients discontinued bedaquiline due to AEs. Post-baseline abnormal QTcF values > 500 ms were reported in only 2.5% of the 2278 bedaquiline-treated patients with post-baseline ECG data, and most of these patients were also receiving levofloxacin, moxifloxacin or clofazimine, all known to prolong the QT interval. This is consistent with results from two previous single-center observational cohort studies (one prospective, n = 195, and one retrospective, n = 420, respectively) conducted in South Africa, in which 2.2% and 4.3% of patients treated with bedaquiline had a QTcF value > 500 ms respectively [15, 16]. It is worth noting that only a very small proportion of patients in the non-bedaquiline-treated group had available ECG data relative to the bedaquiline-treated group, as this type of monitoring was a recommendation by the 2013 WHO interim guidelines [21] for patients treated with bedaquiline. Reports of AESIs were generally uncommon.

Strengths of the study include the large sample size, supplementation of long-term mortality data from the South African National Vital Statistics Register, real-world use of bedaquiline, a robust analysis including a sensitivity analysis and random selection of participants for the analysis. This study adds to the published literature in terms of real-world treatment outcome and AE data for bedaquiline-treated patients, as well as resistance data, although limited, and a long-term mortality analysis.

As this was an observational study, there are some inherent limitations and considerations when interpreting the data. Firstly, the allocation of study treatment was not random, and this may have resulted in selection bias (e.g. more patients with pre-XDR-TB and XDR-TB were being treated with newer regimens, including bedaquiline as a result of having fewer treatment options, based on recommendations in treatment guidelines). However, attempts were made to adjust for confounding differences in baseline characteristics in the analysis. Data were collected as per routine clinical practice at the various study sites, which could lead to missing data, errors in data collection, and incomplete outcome assessment due to patients being lost to follow-up. There were also no statistical processes in place to compensate for the impact of missing data and this may have affected the accuracy of results where data was missing. However, missing data was only reported in < 1% of patients for critical variables, so any accuracy errors were not expected to be major. Moreover, there may have been differences in completeness of data collection by treatment because of increased monitoring and reporting for bedaquiline-treated patients that was recommended [25]. As the study included all patients newly diagnosed with MDR-TB and those newly treated with bedaquiline, all bedaquiline-treated patients, regardless of (prior) diagnosis, could be included. Toxicity of a previous MDR-TB regimen was one of the main reasons for switching to a bedaquiline-containing regimen, and bedaquiline-treated patients were more likely than non-bedaquiline-treated patients to have been previously treated with second-line MDR-TB drugs, and had longer follow-up. Additionally, non-bedaquiline-treated patients who stopped MDR-TB treatment for any reason and who later started a new MDR-TB treatment could be included in both treatment groups. Although mortality at the end of DR-TB treatment was captured as a WHO treatment outcome in EDRWeb for which data completeness was included as part of the medical record abstraction process, long-term mortality collected based on data supplemented from the Vital Statistics Register were less complete, as only those patient records with a valid South African national identification number could be matched with the Vital Statistics Register. A higher proportion of DR-TB registration numbers could not be matched in the non-bedaquiline-treated group (588/2237 [26%]) than in the bedaquiline-treated group (515/3765 [14%]), so potentially fewer outcomes were observed in the comparator group for long-term mortality. There is also the potential for survivorship bias (partially based on when bedaquiline was initiated in the treatment course) where patients had to survive long enough and not be lost to follow-up, to initiate and receive bedaquiline treatment. Finally, there was a difference between the number of bedaquiline-treated patients who had a positive sputum culture in EDRWeb (n = 2483) compared to patients with a valid sample for DST by the NICD (n = 383) at baseline, due to differences in the processing of samples by the NICD compared with the local laboratories.

In conclusion, the data from this large, longitudinal cohort study of MDR-TB patients in South Africa provide real-world evidence indicating that the addition of bedaquiline to treatment regimens was associated with a survival and effectiveness benefit compared with treatment regimens without bedaquiline. The acquisition of resistance to bedaquiline while on therapy was low (3.5% of evaluable isolates), although continued monitoring of bedaquiline resistance is recommended. No new safety signals for bedaquiline were detected. The observed effectiveness and safety are consistent with the positive risk–benefit profile of bedaquiline and support continued implementation as part of combination therapy for the treatment of pulmonary MDR-TB.

## Supplementary Information

**Additional file 1: Table S1.** Summary of treatment guidelines in South Africa during the Registry period**. Table S2.** Pre-2021 Treatment outcome definitions used in this study. **Table S3.** Patient disposition in the South African Registry study. **Table S4.** Propensity score sensitivity analysis for risk of mortality (EDRWeb data only, end of treatment). **Figure S1.** Effect of a bedaquiline-based regimen on **A** treatment success and **B** mortality (EDRWeb data only, end of treatment) for patient subgroups in the South African Registry study. **Figure S2.** Long-term follow-up mortality (up to 30 months after MDR-TB treatment start, including data from the South African National Vital Statistics Register): Kaplan-Meier survival curves for **A** the overall study population, **B** MDR-TB-, **C** pre-XDR-TB- and **D** XDR-TB-infected patients in South Africa treated with bedaquiline- or non-bedaquiline-containing regimens.

## Data Availability

The data presented in this manuscript are from EDRWeb, a national electronic register of South African DR-TB patients receiving treatment, which is owned by the South African National Department of Health (NDoH), Dr AB Xuma Building, 1112 Voortrekker Rd, Pretoria, Townlands 351-JR, Pretoria 0187. P/bag × 828 Pretoria 0001, South Africa. Access to the data was by consent of the NDoH.
