# Supplementary material for: Bedaquiline safety, efficacy, utilization and emergence of resistance following treatment of multidrug-resistant tuberculosis patients in South Africa: a retrospective cohort analysis
Source: BMC Infect Dis. 2022 Nov 21;22:870. doi: 10.1186/s12879-022-07861-x (PMC9682840; doi:10.1186/s12879-022-07861-x)
Supplement: Supplementary file 1 — Additional file 1: Table S1. Summary of treatment guidelines in South Africa during the Registry period. Table S2. Pre-2021 Treatment outcome definitions used in this study. Table S3. Patient disposition in the South African Registry study. Table S4. Propensity score sensitivity analysis for risk of mortality (EDRWeb data only, end of treatment). Figure S1. Effect of a bedaquiline-based regimen on A treatment success and B mortality (EDRWeb data only, end of treatment) for patient subgroups in the South African Registry study. Figure S2. Long-term follow-up mortality (up to 30 months after MDR-TB treatment start, including data from the South African National Vital Statistics Register): Kaplan-Meier survival curves for A the overall study population, B MDR-TB-, C pre-XDR-TB- and D XDR-TB-infected patients in South Africa treated with bedaquiline- or non-bedaquiline-containing regimens. [file 12879_2022_7861_MOESM1_ESM.docx]

**Bedaquiline safety, efficacy, utilization and emergence of resistance following treatment of multidrug-resistant tuberculosis patients in South Africa: a retrospective cohort analysis**

Helen Pai^1^, Norbert Ndjeka^2^, Lawrence Mbuagbaw^3^, Koné Kaniga^4^, Eileen Birmingham^1^, Gary Mao^4^, Lori Alquier^1^, Kourtney Davis^4^, Arianne Bodard^5^, Abeda Williams^6^, Magalie Van Tongel^5^, Florence Thoret-Bauchet^7^ and Nyasha Bakare^4^

^1^Janssen Research and Development, LLC, Raritan, NJ, USA; ^2^National TB Programme, South African National Department of Health, Pretoria, South Africa; ^3^ Department of Health Research Methods, Evidence and Impact, McMaster University, Hamilton, Ontario, Canada; ^4^Janssen Research & Development, LLC, Titusville, NJ, USA; ^5^Janssen Pharmaceutica NV, Beerse, Belgium; ^6^Janssen Pharmaceutica (Pty.) Ltd, Johannesburg, South Africa; ^7^Janssen-Cilag, Issy-les-Moulineaux, France.

**Additional Section**

**Table S1.** Summary of treatment guidelines in South Africa during the Registry period

|  | **Intensive treatment phase** | **Continuation Phase** |
| --- | --- | --- |
| **2011 National Treatment Guidelines** | | |
| MDR-TB | 6 months KM-MFX-ETO-TRD-PZA | Up to 18 months after conversion  MFX-ETO-TRD-PZA |
| XDR-TB | 6 months  CM-MFX-ETO-TRD-PZA-PAS-CFZ | Up to 18 months after conversion  MFX-ETO-TRD-PZA-PAS-CFZ |
| **2017 National Treatment Guidelines** | | |
| MDR-TB (short-course) | 4 months  KM-MFX-CFZ-ETO-INH-EMB-PZA | Up to 5 months after conversion  MFX-CFZ-ETO-PZA |
| MDR-TB (long-course) | 6 to 8 months  KM-MFX-ETO-TRD-PZA | Up to 12 months after conversion  MFX-ETO-TRD-PZA |
| XDR-TB | 6 to 8 months  Bedaquiline-TRD-CFZ-LZD-LFX | Up to 12 months after conversion  TRD-CFZ-LZD-LFX |

MDR-TB, multidrug-resistant-tuberculosis, defined as *Mycobacterium tuberculosis* strains that are resistant to at least isoniazid and rifamp(ic)in.

XDR-TB, extensively drug resistant-tuberculosis, defined as MDR-TB with additional resistance to a fluoroquinolone and a second line injectable drug.

CM, capreomycin; CFZ, clofazimine; EMB, ethambutol; ETO, ethionamide; INH, isoniazid; KM, kanamycin; LFX, levofloxacin; LZD, linezolid; MFX, moxifloxacin; PAS, para-aminosalicylic acid;
PZA, pyrazinamide; TRD, terizidone

When bedaquiline was not yet included in the standard MDR-TB treatment regimen, bedaquiline was recommended for addition to the regimen for pre-XDR-TB and XDR-TB patients, or to replace amikacin, capreomycin or kanamycin when any level of hearing loss was detected as recommended in the 2015 South African policy guideline for introduction of new drugs and drug regimens.^1^

Following the introduction of the shorter MDR-TB regimen by the WHO in 2016,^2^ the South African national DR-TB treatment guidelines were updated in 2017 to include the new short-course MDR-TB treatment regimen for patients not previously exposed to second-line TB agents with a *katG* or *inhA* mutation. However, none of the patients in the registry received the short-course regimen.

**Table S2** Pre-2021 Treatment outcome definitions used in this study

| **Outcome** | **Definition** |
| --- | --- |
| Cured | “A pulmonary TB patient with bacteriologically confirmed TB at the beginning of treatment who was smear- or culture-negative in the last  month of treatment and on at least one previous occasion.” |
| Treatment completed | A TB patient who completed treatment without evidence of failure BUT with no record to show that sputum smear or culture results in the last  month of treatment and on at least one previous occasion were negative, either because tests were not done or because results are unavailable. |
| Treatment failed | “A TB patient whose sputum smear or culture is positive at month 5 or later during treatment.” |
| Died | “A TB patient who dies for any reason before starting or during the course of treatment.” |
| Lost to follow-up | “A TB patient who did not start treatment or whose treatment was interrupted for 2 consecutive months or more.” |
| Not evaluated | **“**A TB patient for whom no treatment outcome is assigned. This includes cases “transferred out” to another treatment unit as well as cases for whom the treatment outcome is unknown to the reporting unit.” |
| Treatment success | “The sum of cured and treatment completed.” |

**Table S3** Patient disposition in the South African Registry study

|  | **Bedaquiline  N=3,747** | **No bedaquiline N=2,234** | **Total  N=5,981** |
| --- | --- | --- | --- |
| Safety population,^a^ n (%) | 3,747 (100) | 2,234 (100) | 5,981 (100) |
| Evaluable population,^b^ n (%) | 3,739 (99.8)^c^ | 2,231 (99.9)^c^ | 5,970 (99.8)^c^ |
| Patients evaluated for baseline DST, n (%) | 3,172 (84.7) | 1,567 (70.1) | 4,739 (79.2) |
| Bedaquiline-treated NICD set, n (%) | 383 (10.2) | N/A | N/A |
| Duration of follow-up,^d^ months | **N=3,743** | **N=2,231** | **N=5,974** |
| Mean (SD) | 18.6 (7.5) | 14.8 (9.1) | 17.2 (8.3) |
| Median (range) | 20.5 (0; 46.1) | 18.2 (0; 49.7) | 19.8 (0; 49.7) |
| Number of follow-up visits^e^ |  |  |  |
| Mean (SD) | 18.9 (12.1) | 9.2 (7.0) | 15.3 (11.5) |
| Median (range) | 17.0 (0; 81.0) | 8.0 (0; 63.0) | 13.0 (0; 81.0) |
| NICD, National Institute for Communicable Diseases; N/A, not applicable; SD, standard deviation  ^a^The safety population includes all patients who were registered in EDRWeb and who were exposed to at least one dose of bedaquiline or non-bedaquiline treatment during the study  ^b^The evaluable population includes all patients in the safety population who have baseline and at least one postbaseline clinical or/and microbiological assessment  ^c^The 11 patients excluded from the evaluable population were four patients that were still on treatment (all bedaquiline-treated) and seven patients with missing treatment outcome (four bedaquiline-treated patients and three non-bedaquiline-treated patients)  ^d^Duration of follow-up is defined as the months from the treatment start date to EDRWeb outcome date  ^e^Based on the number of visits for sputum samples (only one sample counted per day) | | | |

**Table S4** Propensity Score Sensitivity Analysis for Risk of Mortality (EDRWeb data only, end of treatment)

| **Propensity score analysis** | **Hazard ratio  (95% confidence interval)** |
| --- | --- |
| Matched on propensity score | 0.46 (0.41, 0.52) |
| Adjusted for propensity score deciles | 0.34 (0.30, 0.39) |
| Inverse probability weighting | 0.51 (0.45, 0.57) |

**
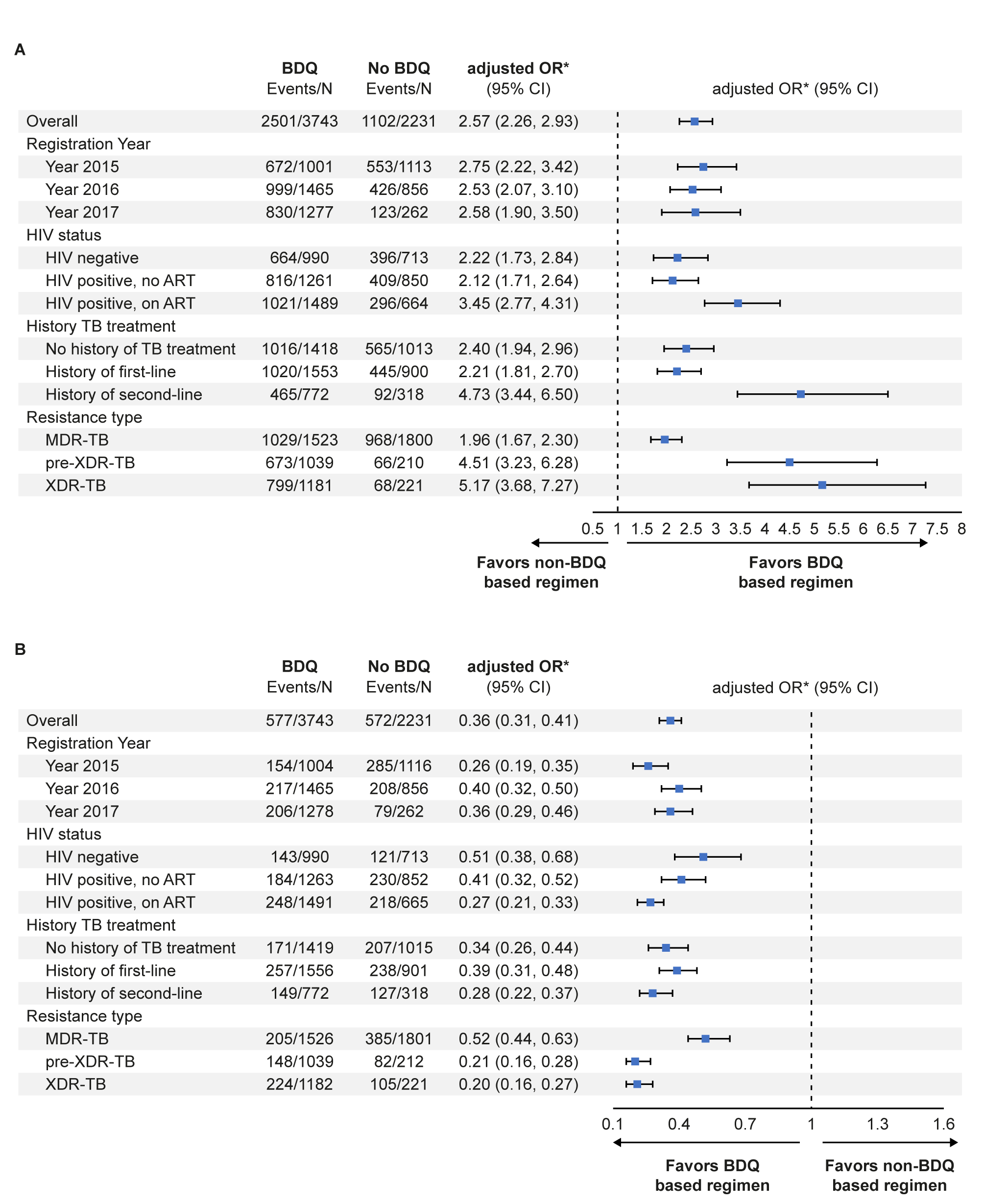
**

*Odds ratios and hazard ratios adjusted for: gender (male, female), age, province (Eastern Cape, Gauteng, KwaZulu-Natal, Western Cape), HIV Status (negative, positive not on ART, positive with ART started), type of TB (pulmonary, extrapulmonary), previous drug history (No history of TB treatment, history of first-line TB treatment; history of second-line TB treatment), registration year (2015, 2016, 2017), DR-TB Type (MDR, XDR, pre-XDR) except for the specific subgroup of interest

The total N within each subgroup analysis may not sum to BDQ total (N=3,747) and non-BDQ total (N=2,234) due to missing observations; Treatment success includes treatment completed or cured; Mortality assessed as death per WHO treatment outcome as recorded in EDRWeb

**Figure S1** Effect of a bedaquiline-based regimen on **A** treatment success and **B** mortality (EDRWeb data only, end of treatment) for patient subgroups in the South African Registry study

**
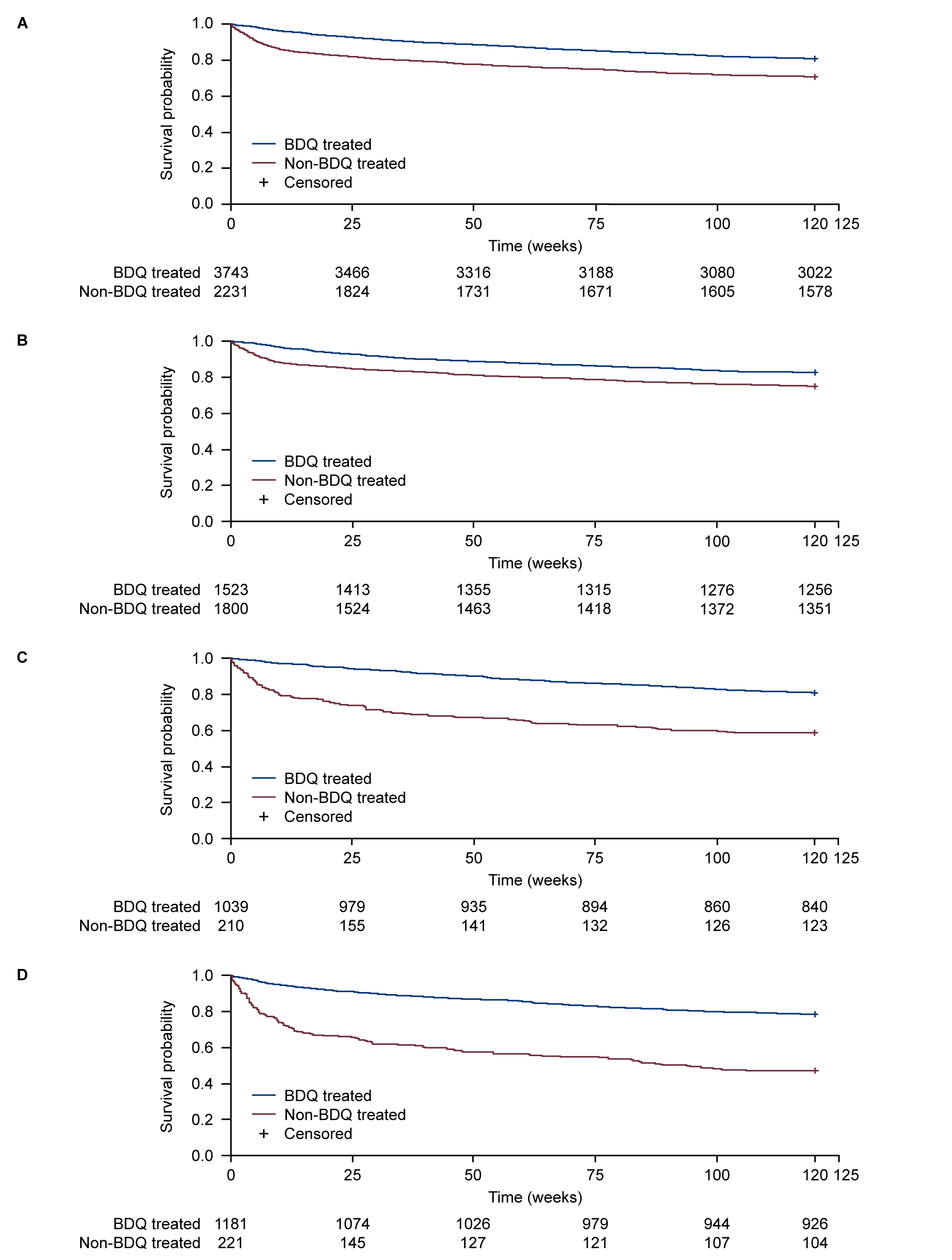
**

**Figure S2** Long-term follow-up mortality (up to 30 months after MDR-TB treatment start, including data from the South African national Vital Statistics Register): Kaplan-Meier survival curves for **A** the overall study population, **B** MDR-TB-, **C** pre-XDR-TB- and **D** XDR-TB-infected patients in South Africa treated with bedaquiline- or non-bedaquiline-containing regimens

**References**

1. Directorate Drug-Resistant TB, TB & HIV. Introduction of new drugs, drug regimens and management for drug-resistant TB in South Africa: Policy Framework. 1.1. Pretoria: National Department of Health; 2015.
2. World Health Organization. The Shorter MDR-TB Regimen. May 2016. Available at: http://www.who.int/tb/Short_MDR_regimen_factsheet.pdf. Accessed October 15 2020.STROBE Statement—checklist of items that should be included in reports of observational studies

|  | **Item No.** | **Recommendation** | **Page  No.** | **Relevant text from manuscript** |
| --- | --- | --- | --- | --- |
| **Title and abstract** | 1 | (*a*) Indicate the study’s design with a commonly used term in the title or the abstract | 1 and 2 | Title and abstract: ‘…retrospective cohort analysis.’ |
|  |  | (*b*) Provide in the abstract an informative and balanced summary of what was done and what was found | 2 and 3 | See text on pages 2 and 3 |
| **Introduction** | | | |  |
| Background/rationale | 2 | Explain the scientific background and rationale for the investigation being reported | 3 to 5 | Background: see page 3 and 4 Rationale: ‘A post-marketing requirement for the accelerated approval of SIRTURO® by the United States Food and Drug Administration (FDA) included the development of a patient registry for bedaquiline-treated patients. Additionally, the 2013 WHO interim policy guidance on the use of bedaquiline in the treatment of MDR-TB [17] identified the need for collection of additional data from larger patient populations beyond data provided from the bedaquiline Phase 2 clinical development program.’ |
| Objectives | 3 | State specific objectives, including any prespecified hypotheses | 5 | ‘Here we report the safety, effectiveness, drug utilization and emergence of resistance associated with the use of bedaquiline in the treatment of MDR-TB using data from EDRWeb.’ |
| **Methods** | | | |  |
| Study design | 4 | Present key elements of study design early in the paper | 6 to 8 | See text on pages 6 to 8 under the subheadings ‘The South African registry’ and ‘Study design and patients’ |
| Setting | 5 | Describe the setting, locations, and relevant dates, including periods of recruitment, exposure, follow-up, and data collection | 6 to 9 | See text on pages 6 to 9 under the subheadings ‘The South African registry’, ‘Study design and patients’ and ‘Data collection and analysis’ |
| Participants | 6 | (*a*) *Cohort study*—Give the eligibility criteria, and the sources and methods of selection of participants. Describe methods of follow-up  *Case-control study*—Give the eligibility criteria, and the sources and methods of case ascertainment and control selection. Give the rationale for the choice of cases and controls  *Cross-sectional study*—Give the eligibility criteria, and the sources and methods of selection of participants | 6 to 9 | See text on pages 6 to 9 under the subheadings ‘The South African registry’ and ‘Study design and patients’ |
|  |  | (*b*) *Cohort study*—For matched studies, give matching criteria and number of exposed and unexposed  *Case-control study*—For matched studies, give matching criteria and the number of controls per case | 7 and 12 | See text on page 7 under the subheading ‘Study design and patients’  ‘All patients newly diagnosed with MDR-TB (including pre-XDR-TB and XDR-TB and excluding RR-TB) and those newly treated with bedaquiline at participating sites, were eligible for inclusion in the MDR-TB registry. No specific exclusion criteria were applied in this study (so that patients could have been previously treated for TB). A randomly selected subset of MDR-TB patients not exposed to bedaquiline (the comparator cohort) was selected from either patients who started treatment in the 6-month period prior to bedaquiline availability in South Africa or, once bedaquiline became available, from those prescribed a non-bedaquiline-containing regimen.’  See text on page 12 under the subheading ‘Patient disposition’  ‘Data was extracted from EDRWeb for 5,981 patients with MDR-TB, exposed to at least one dose of the bedaquiline-containing (N=3,747) or non-bedaquiline-containing regimen (N=2,234) (safety population) at 14 study sites in South Africa between 2015 and 2017.’ |
| Variables | 7 | Clearly define all outcomes, exposures, predictors, potential confounders, and effect modifiers. Give diagnostic criteria, if applicable | 8 to 10 | See text on page 8 to 10 under the subheading: ‘Data collection and analysis’  Especially see pages 9 and 10:  ‘As patients were not randomly assigned to the treatment groups, comparisons of outcomes between treatment groups might be confounded by selection bias. Therefore, treatment success was compared between treatment groups using multivariable logistic regression analysis, adjusting for gender, age, province, human immunodeficiency virus (HIV) status, site of TB (pulmonary or extrapulmonary), history of TB treatment, year of patient registration in EDRWeb and DR-TB type, to estimate the odds ratios (ORs) and 95% CIs for the association between the use of a bedaquiline-containing regimen and treatment success.’  ‘A multivariable Cox proportional hazards model was used to estimate the hazard ratios (HRs) and 95% CIs for the association between the use of a bedaquiline-containing regimen (compared with a non-bedaquiline-containing regimen) and the risk of mortality, adjusting for potential confounders based on the baseline clinical characteristics and patient demographics described above.’  ‘In addition, propensity score (PS) methods were used to adjust for imbalances in demographic and disease characteristics between the groups using logistic regression to model the probability of receiving bedaquiline treatment conditional on the potential confounding variables measured and available in the data set.’ |
| Data sources/ measurement | 8* | For each variable of interest, give sources of data and details of methods of assessment (measurement). Describe comparability of assessment methods if there is more than one group | 8 to 12 | See text on page 8 to 12 under the subheading: ‘Data collection and analysis’ |
| Bias | 9 | Describe any efforts to address potential sources of bias | 8 to 10 | See answer to item #7 above. |
| Study size | 10 | Explain how the study size was arrived at | 8 | See text on page 8:  ‘Based on sample size estimates, a registry of 1,000 bedaquiline-exposed patients per year over 3 years would have 95% power to detect an adverse event (AE) with a relative risk of 2, assuming alpha = 0.05 and a 1:1 ratio of bedaquiline-exposed patients to unexposed patients, for events that occur with a frequency of at least 3%.’ |
| Quantitative variables | 11 | Explain how quantitative variables were handled in the analyses. If applicable, describe which groupings were chosen and why | 8 to 12 | See text on page 8 to 12 under the subheading: ‘Data collection and analysis’ |
| Statistical methods | 12 | (*a*) Describe all statistical methods, including those used to control for confounding | 8 to 12 | See answer to item #7 above. |
|  |  | (*b*) Describe any methods used to examine subgroups and interactions | 10 | See text on page 10:  ‘A subgroup analysis for treatment success and risk of mortality was performed by year of registration in EDRWeb, gender, age, HIV status, previous drug history and DR-TB type.’ |
|  |  | (*c*) Explain how missing data were addressed | 10 and 12 | See text on page 10:  ‘Missing data was unavoidable due to the retrospective nature of the study. Efforts were made to minimize missing data via medical record abstraction and matching to the national Vital Statistics Register for mortality. Counts of patients with missing data were reported in all analyses where appropriate (Table 1, Table S3) and were less than 1% for critical variables; therefore, no imputation was performed.”  Page 12: ‘Eleven patients were excluded from the evaluable population as they were still on treatment (four bedaquiline-treated patients) or had a missing treatment outcome (four bedaquiline-treated patients and three non-bedaquiline-treated patients) at the time of data extraction.’ |
|  |  | (*d*) *Cohort study*—If applicable, explain how loss to follow-up was addressed  *Case-control study*—If applicable, explain how matching of cases and controls was addressed  *Cross-sectional study*—If applicable, describe analytical methods taking account of sampling strategy | 9 and 11 | See text on Page 9:  ‘Treatment outcome (cured, treatment completed, treatment failed, died, lost to follow-up, not evaluated and treatment success [i.e. the sum of cured and treatment completed patients]), as reported in EDRWeb according to standard WHO definitions pre-2021 (Table S2) [22], within 6 months after end-of-treatment was assessed among bedaquiline- and non-bedaquiline-treated MDR-TB patients.’  Page 11:  ‘Long-term mortality data (up to 30 months after the start of treatment) was retrieved from the South African national Vital Statistics Register (for patients lost to follow-up, with missing WHO treatment outcome in EDRWeb, or with deaths reported after the WHO treatment outcome).’ |
|  |  | (*e*) Describe any sensitivity analyses | 10 | See text on Page 10:  ‘Multiple statistical approaches using the PS were applied in Cox models, including matching, stratification by PS decile, and inverse probability of treatment weighting. These PS statistical approaches were conducted as a sensitivity analysis to the primary analysis method of multivariable Cox proportional hazards regression for the adjusted association between bedaquiline-containing regimens and death.’ |
| **Results** | | | | |
| Participants | 13* | (a) Report numbers of individuals at each stage of study—eg numbers potentially eligible, examined for eligibility, confirmed eligible, included in the study, completing follow-up, and analysed | 12 | See text on Page 12:  ‘Data was extracted from EDRWeb for 5,981 patients with MDR-TB, exposed to at least one dose of the bedaquiline-containing (N=3,747) or non-bedaquiline-containing regimen (N=2,234) (safety population) at 14 study sites in South Africa between 2015 and 2017. Of the 5,981 patients, 5,970 (99.8%) patients had baseline and at least one post-baseline WHO treatment outcome and/or microbiological assessment (evaluable population). Eleven patients were excluded from the evaluable population as they were still on treatment (four bedaquiline-treated patients) or had a missing treatment outcome (four bedaquiline-treated patients and three non-bedaquiline-treated patients) at the time of data extraction. Full details of the patient disposition in the South African Registry are presented in Table S3.’ |
|  |  | (b) Give reasons for non-participation at each stage | 12 | See previous answer. |
|  |  | (c) Consider use of a flow diagram |  | See Table S3. |
| Descriptive data | 14* | (a) Give characteristics of study participants (eg demographic, clinical, social) and information on exposures and potential confounders | 12 and 13 | See text on page 12 and 13 under the subheading: ‘Patient disposition’ and also Table 1 |
|  |  | (b) Indicate number of participants with missing data for each variable of interest | 12 | See text on page 12: ‘Eleven patients were excluded from the evaluable population as they were still on treatment (four bedaquiline-treated patients) or had a missing treatment outcome (four bedaquiline-treated patients and three non-bedaquiline-treated patients) at the time of data extraction. Full details of the patient disposition in the South African Registry are presented in Table S3.’  See also Tables 1, 2, 4 and S3 and figure S1. |
|  |  | (c) *Cohort study*—Summarise follow-up time (eg, average and total amount) | 12 | See text on page 12 ‘Mean (standard deviation [SD]) estimated duration of registry follow-up (estimated from treatment startup to treatment outcome in EDRWeb) was 18.6 (7.5) months for the bedaquiline-treated and 14.8 (9.1) months for non-bedaquiline-treated patients. The total person-time follow-up was 3,775 and 1,681 person-years, respectively.’  See also Table S3. |
| Outcome data | 15* | *Cohort study*—Report numbers of outcome events or summary measures over time |  | All results and figures/tables depict these results.  Especially over time, Figure 1 shows the Kaplan-Meier survival curve for deaths defined per WHO Treatment Outcome and Figure S2 shows long-term follow-up mortality (up to 30 months after MDR-TB treatment start, including data from the national Vital Statistics Register) |
|  |  | *Case-control study—*Report numbers in each exposure category, or summary measures of exposure |  | Not applicable |
|  |  | *Cross-sectional study—*Report numbers of outcome events or summary measures |  | Not applicable |
| Main results | 16 | (*a*) Give unadjusted estimates and, if applicable, confounder-adjusted estimates and their precision (eg, 95% confidence interval). Make clear which confounders were adjusted for and why they were included | 15 to 20 | See text on pages 15 to 20 under the subheadings: ‘WHO treatment outcome’ and ‘Long-term follow-up mortality data’. |
|  |  | (*b*) Report category boundaries when continuous variables were categorized |  | Not applicable |
|  |  | (*c*) If relevant, consider translating estimates of relative risk into absolute risk for a meaningful time period |  | Not applicable |
| Other analyses | 17 | Report other analyses done—eg analyses of subgroups and interactions, and sensitivity analyses | 16, 19 and 20 | See Page 16: ‘Numerically higher proportions of bedaquiline- versus non-bedaquiline-treated patients with MDR-TB, pre-XDR-TB and XDR-TB achieved treatment success (Table 3).’  See also page 19:  ‘In general, a consistent beneficial effect of a bedaquiline-containing regimen on treatment success was observed across subgroups (Figure S1a).’  ‘The effect of a bedaquiline-containing regimen on reducing the risk of mortality was also consistent across the various subgroups (Figure S1b).’  ‘The results of the various propensity score statistical approaches from sensitivity analyses were consistent with the findings from the adjusted Cox proportional hazards model, showing a consistent beneficial effect of bedaquiline-containing regimens on the risk of mortality (WHO treatment outcome) (Table S4).’  See also page 19 to 20:  ‘Mortality at 30 months (130 weeks) was reported in 721/3,743 (19.3%) bedaquiline-treated and 654/2231 (29.3%) non-bedaquiline-treated patients, with the multivariable Cox proportional hazards analysis showing a decreased risk of long-term mortality for bedaquiline-treated compared with non-bedaquiline-treated patients overall (HR 0.43; 95% CI: 0.38, 0.49) and across subgroups (data not shown).’  See also Table S4 and figure S1. |
| **Discussion** | | | | |
| Key results | 18 | Summarise key results with reference to study objectives | 23 to 24 | See paragraph on page 23 to 24:  ‘This retrospective, observational cohort study using data from the South African national patient register of DR-TB patients showed that treatment success (sum of cured and treatment completed patients) was achieved in 66.9% of bedaquiline-treated and 49.4% of non-bedaquiline-treated patients, and death was reported in 15.4% and 25.6% of patients, respectively. Adjusted multivariable Cox proportional hazards analyses showed that treatment with a bedaquiline-containing regimen was associated with increased likelihood of treatment success and decreased risk of short- and long-term mortality compared with an MDR-TB regimen not containing bedaquiline. Furthermore, a consistent beneficial effect of a bedaquiline-containing regimen on treatment success and mortality risk was observed across the subgroups. However, some subgroups included a limited number of patients, with an imbalance in the proportion of bedaquiline-treated and non-bedaquiline-treated patients, so the results should be interpreted with caution. The majority of TEAEs generally reflected the known safety profile of bedaquiline and background regimen medications.’ |
| Limitations | 19 | Discuss limitations of the study, taking into account sources of potential bias or imprecision. Discuss both direction and magnitude of any potential bias | 26 to 28 | See page 26 to 28, paragraph starting:  ‘As this was an observational study, there are some inherent limitations and considerations when interpreting the data. Firstly, the allocation of study treatment was not random, and this may have resulted in selection bias (e.g. more patients with pre-XDR-TB and XDR-TB were being treated with newer regimens, including bedaquiline as a result of having fewer treatment options, based on recommendations in treatment guidelines)…’ |
| Interpretation | 20 | Give a cautious overall interpretation of results considering objectives, limitations, multiplicity of analyses, results from similar studies, and other relevant evidence | 23 to 28 | See text on pages 30 and 31 |
| Generalisability | 21 | Discuss the generalisability (external validity) of the study results | 26 to 28 | See text on pages 29 to 31 |
| **Other information** | |  | | |
| Funding | 22 | Give the source of funding and the role of the funders for the present study and, if applicable, for the original study on which the present article is based | 30 | See text on page 30 under the subheading ‘Acknowledgements and disclosures’  ‘… We would also like to thank the study centre staff and all Janssen study personnel. This study was sponsored by Janssen Research & Development...’ |

*Give information separately for cases and controls in case-control studies and, if applicable, for exposed and unexposed groups in cohort and cross-sectional studies.
